# Editorial: Community series in the regulation of the host immune activation in respiratory virus infections, volume II

**DOI:** 10.3389/fimmu.2025.1695366

**Published:** 2025-09-26

**Authors:** Alak Manna, Chung-Guei Huang, Mitali Chatterjee, Ching-Tai Huang, Avijit Dutta

**Affiliations:** ^1^ Mayo Clinic, Jacksonville, FL, United States; ^2^ College of Medicine, Chang Gung University, Taoyuan, Taiwan; ^3^ Department of Laboratory Medicine, Chang Gung Memorial Hospital, Chang Gung Medical Foundation, Taoyuan, Taiwan; ^4^ Department of Pharmacology, Institute of Post Graduate Medical Education and Research, Kolkata, India; ^5^ Division of Infectious Diseases, Department of Medicine, Chang Gung Memorial Hospital, Chang Gung Medical Foundation, Taoyuan, Taiwan; ^6^ Amity Institute of Virology and Immunology, Amity University Uttar Pradesh, Gautam Budha Nagar District, Uttar Pradesh, India

**Keywords:** respiratory virus infection, host immune response, regulation of host immunity, community series, editorial

Respiratory viruses cause serious emerging and re-emerging infectious diseases. Not only do the catastrophic novel influenza viruses, SARS-CoV-1, MERS-CoV, and SARS-CoV-2 pose threats, but seasonal influenza viruses periodically lead to significant illness and death https://www.who.int/news-room/fact-sheets/detail/influenza-(seasonal). The host immune system responds to infection in an effort to eliminate the pathogen. However, dysregulated or overwhelming immune responses are thought to be major causes of severe illness and death. “Cytokine storm” has been observed in SARS, severe influenza, and COVID-19 (1–3). Key disease mechanisms in COVID-19, like ADE (Antibody-Dependent Enhancement) and HITT (Heparin-Induced Thrombocytopenic Thrombosis), also stem from immune system dysregulation (4, 5). The adaptive immune system, including T cells, plays a central role here, but the precise mechanisms remain unclear. Balancing protective immunity with controlled inflammation is a major challenge, as attempts to suppress immune overactivation with broad-spectrum immunosuppressants often fall short (6, 7). These agents can hinder the immune system’s ability to clear viruses while suppressing inflammation, sometimes worsening the disease (8). A detailed understanding of immune activation and regulation during severe respiratory virus infections is essential for improving and tailoring treatments.

We have published two volumes in this community series, both of which focus on recent advancements in understanding immune activation and regulation during respiratory virus infections. The series encompasses a range of research on immune regulation, viral evasion, biomarkers, and novel therapies, providing valuable insights into respiratory viruses, host immunity, and their intricate interactions. Among these studies, Li et al. identified a novel immune evasion strategy used by African swine fever virus (ASFV), where the viral protein pA151R degrades the E3 ligase TRAF6, suppressing type I interferon production. Similarly, Farooq Rashid reviewed immune evasion mechanisms by influenza A viral proteins, shedding light on how viruses weaken host immune responses. Intercellular communication also plays a critical regulatory role. Harvey et al. demonstrated that interactions between dendritic cells and natural killer cells influence T cell activation during influenza A infection, highlighting the importance of innate-adaptive immune crosstalk. Huang et al. provided mechanistic insights into how the STING pathway promotes neutrophil extracellular traps (NETs) via GSDMD activation in influenza-associated pneumonia. This work connects cytotoxic inflammation with DNA-sensing pathways in innate immunity, identifying potential targets to reduce lung injury in severe viral pneumonia. Frédéric Rivière examined the wave-like recruitment of innate immune cells in a murine influenza model, providing insights into immune infiltration patterns and their correlation with tissue damage. In another example, Geffen et al. showed how myeloid-derived suppressor cells (MDSCs) contribute to influenza-triggered asthma exacerbation, illustrating how respiratory viruses can worsen chronic inflammatory conditions. Yu et al. explored COVID-19–induced autoimmune hepatitis, demonstrating how SARS-CoV-2 infection and resultant immune dysregulation provoke autoimmune effects. Such dysregulation often leads to the release of soluble immune checkpoint molecules (sICMs) in COVID-19 patients, as reported by Paranga et al. Notably, sICM profiles vary across infections with different SARS-CoV-2 variants and are linked to disease severity and mortality. Huapaya et al. found that alterations in the plasma proteome persist for at least ten months after recovery from mild to moderate COVID-19, emphasizing how immune and metabolic disturbances contribute to post-acute sequelae of SARS-CoV-2 infection (PASC) and “long COVID.”

Host intrinsic factors also influence immune responses. For example, Fomin et al. reported that aging and a high-fat diet worsen lung inflammation in SARS-CoV-2-infected hamsters. Similarly, Sasipa Tanyaratsrisakul identified increased expression of the type 2 asthma-associated molecule MYADM after human rhinovirus infection. These findings suggest viral infections may trigger or worsen allergic airway diseases. The disease course can also be affected by the host’s metabolic status and environmental factors. Bishop et al. showed that exposure to microplastics in the lungs worsens innate immune dysregulation during SARS-CoV-2 infection.

All the manuscripts in these two volumes of the series support a simple message: orchestrated responses of innate and adaptive immunity reduce pathology and promote better recovery from respiratory viral infections. A prime example is the study by Jordi Rodon et al., which offers valuable insights into natural host-virus coevolution and tolerance mechanisms. In camelids, MERS-CoV sensing leads to coordinated innate and adaptive immune responses with minimal tissue damage. While T cells play a significant role, vaccine-induced T cell immunity also provides post-exposure protection in SARS-CoV-2 infection. The study by Somogyi et al. presented an alternative to antibody-focused vaccine strategies.

This community series also published translationally relevant preclinical studies with potential therapeutic perspectives. Silvia Martínez-Diz et al. identified elevated levels of TMPRSS2, CD163/CD206, and CD33 as potential biomarkers for COVID-19 severity. In a SARS-CoV-2-infected hamster model, Afsal Kolloli et al. demonstrated a reduction of lung inflammation and fibrosis with the treatment of a phosphodiesterase-4 inhibitor. Empiric usage does not always yield prospective results. Curran et al. examined anti-PD-L1 therapy in a murine coronavirus pneumonia model. The therapy altered inflammation, but failed to improve survival. It emphasizes the complexity of immunotherapy timing and context during acute infections. Such an intriguing philosophy could also be found in the review by Anthony Bosco, where the emerging roles of interferons in asthma and respiratory infections have been reviewed. The study illustrates how these critical cytokines can either protect against or contribute to pathology depending on the timing and host context.

Collectively, the publications of this Community Series illustrate the importance of timely regulation of immune activation in achieving improved clinical outcomes for certain respiratory viral infections. Cytokine storm with overwhelming and deregulated immune activation is a wider problem during virus infections and is not limited to the respiratory pathogens listed in this Research Topic. Infections of Dengue (9), Zika (10), EBV or cytomegalovirus (11), HHV-8 (also known as Kaposi’s sarcoma herpesvirus) (12), Hep B (13), and many other viruses fall in this category. The quality, magnitude, and kinetics of immune activation are as crucial as the timely regulation of the activation in infectious diseases. Immune activation is required for the clearance of an infection, and this activation should subside with pathogen eradication. It is challenging to distinguish between excessive and appropriate cytokine production for controlling a widespread infection. The magnitude and kinetics should be analyzed thoroughly before devising handles for immune manipulation, to minimize undesired outcomes of inappropriate manipulation.
